# The Therapeutic Effects of Goreisan, a Traditional Japanese Herbal Medicine, on Lower-Limb Lymphedema after Lymphadenectomy in Gynecologic Malignancies: A Case Series Study

**DOI:** 10.1155/2020/6298293

**Published:** 2020-04-17

**Authors:** Nobuhisa Yoshikawa, Hiroaki Kajiyama, Naoki Otsuka, Satoshi Tamauchi, Yoshiki Ikeda, Kimihiro Nishino, Kaoru Niimi, Shiro Suzuki, Fumi Utsumi, Kiyosumi Shibata, Fumitaka Kikkawa

**Affiliations:** ^1^Department of Obstetrics and Gynecology, Nagoya University Graduate School of Medicine, Nagoya 4668550, Japan; ^2^Department of Obstetrics and Gynecology, Fujita Health University Bantane Hospital, Nagoya 4548509, Japan

## Abstract

**Background:**

Lower-limb lymphedema (LLL) is a chronic and progressive complication of gynecologic cancer treatment, including pelvic lymphadenectomy. This study aims to investigate the therapeutic effect of goreisan, a traditional Japanese medicine, which has been used for hydrostatic modulation on patients with LLL.

**Methods:**

Patients diagnosed with LLL in our hospital in 2018 were included and principally treated with complex decongestive therapy (CDT), including elastic clothing and lymph drainage. The patients who received a combination therapy of CDT and goreisan (CDT-G group) were prescribed goreisan extract granules, with a dose of 7.5 g per os daily in three doses. Patients who were not prescribed goreisan received CDT alone (CDT group). The severity of lymphedema was evaluated by the estimated limb volume calculated by limb circumferences and the ratio of extracellular water (ECW) to total body water (TBW).

**Results:**

Nineteen women with LLL after pelvic lymphadenectomy were included in the study. The number of patients in the CDT and CDT-G groups was 8 and 11, respectively. There were no statistically significant differences between the CDT and CDT-G groups in terms of patient characteristics and severity of LLL before treatment. Reduction in ECW/TBW in the CDT-G group (in the whole body and the affected lower limb) after the intervention was significantly more remarkable than that in the CDT group.

**Conclusions:**

Goreisan-based Japanese herbal therapy may be effective in patients with LLL after retroperitoneal lymphadenectomy.

## 1. Introduction

Lower-limb lymphedema (LLL) is one of the most frequent and refractory complications resulting from damage to the lymphatic system after retroperitoneal lymphadenectomy for gynecologic cancer. This postoperative condition is comparatively common after cancer surgery; approximately one-third of patients will be diagnosed as having either lower-limb swelling or LLL [1]. LLL is a symptom of lymphatic insufficiency because of anatomical obstruction following lymphadenectomy. No fundamental approaches to LLL have been developed, and women affected by LLL have no choice but to accept it. In patients with gynecologic cancer, LLL often leads to impairment of quality of life (QOL) in terms of function, emotions, and sexual activity [2, 3]. As the number of patients with cancer who survive for a long time after treatment is increasing, there is a growing need to improve the QOL of patients with cancer suffering from LLL.

The standard treatment for LLL has not been well established. Primarily, patients with LLL are cared for with compression bandaging and manual lymphatic drainage as part of complete decongestive therapy (CDT), which is often cumbersome and leads to patients' frustration [4]. Lymphatic venous anastomosis can be a good option and can theoretically be a fundamental treatment for LLL, but the criteria and procedure have not been well established at this point [5]. Some medicines, including coumarins and flavonoids, have been reported as treatment options for LLL, but the efficacy of these drugs is still controversial and is not standard treatment [6, 7]. Therefore, there is an intensive need to establish a novel treatment for LLL to improve the efficacy of CDT.

Goreisan is one of the traditional Japanese herbal medicines, including “bukuryo” (hoelen), “takusha” (*Alismatis rhizoma*), “sojutsu” (*Atractylodes lanceae rhizoma*), “chorei” (polyporus), and “keihi” (cinnamon bark), and has been widely used in Japan to treat edema, nausea, and headache by modifying interstitial water retention. Goreisan is also widely used in China and Korea, where it is called “wullingsan” and “oryeongsan,” respectively. Goreisan increases urinary output and decreases water retention in the third space, such as diuretics, but its diuretic function does not work in cases of dehydration [8]. Therefore, the biological activity of goreisan is thought to be milder than that of diuretics, and it has few side effects, such as rash and itching. A recent retrospective study reported a 78% effectiveness of combination therapy of goreisan and CDT for LLL with a 2.1-cm median reduction of the abdominal circumference [9]. The efficacy of goreisan for lymphedema has not been fully elucidated, and its efficacy needs to be verified by well-designed prospective studies.

We hypothesized that goreisan combined with CDT would reduce the severity of LLL. In our current study, we investigate the therapeutic effect of goreisan on patients with LLL after retroperitoneal lymphadenectomy for gynecologic cancer.

## 2. Materials and Methods

### 2.1. Patient Enrollment

Women aged 20 years and older who developed LLL in stages I to II, according to the International Society of Lymphology staging system, were enrolled in this study. Between January 2018 and December 2019, we prospectively surveyed a total of consecutive 40 outpatients diagnosed as having unilateral secondary LLL as a complication of treatment for gynecologic cancer at the Nagoya University Hospital in Japan. Patients were eligible for this study if they had adequate heart, lung, kidney, and liver function before enrollment. Patients were excluded from the study if they satisfied any of the following criteria: (1) LLL was before surgery; (2) suspicion of deep venous thrombosis; and (3) a history of use of oral antiedema drugs, such as diuretics or Japanese herbal medicines, for LLL. This study was approved by the Nagoya University Hospital's Ethics Committee. All enrolled participants gave written consent or did not refuse to use their information.

### 2.2. CDT

All patients received CDT for LLL. CDT consisted of simple lymphatic drainage (SLD), compression therapy, and exercise. According to the SLD method, which was reported by De Godoy et al., patients were recommended to conduct SLD by themselves daily [10]. In our hospital, licensed lymphedema therapists perform CDT for all LLL patients. The therapists teach SLD by performing the movements with the patient. Patients were also recommended to wear elastic garments covering the lower extremities and lower abdomen and to keep their skin clear.

### 2.3. Goreisan Administration

Patients who received combination therapy of CDT and goreisan (the CDT-G group) were prescribed goreisan extract granules, a pharmaceutical-grade medicine (Tsumura & Co., Tokyo, Japan) with a dose of 7.5 g per os three times daily. Patients who were not prescribed goreisan received CDT alone (the CDT group).

### 2.4. Measurement and Assessment of Lymphedema

Leg volume, estimated by circumference measurements of the affected limb, and body composition, including the ratio of extracellular water (ECW) to total body water (TBW) (ECW/TBW), were evaluated at baseline and after the 4–15 weeks of intervention. Limb measurements were taken on both legs at seven anatomic locations: (1) the distal side of the first to fifth metatarsal bones; (2) the ankle; (3) the thickest point of the lower limb; (4) the distal side of the kneecap joint; (5) 12 cm proximal from the kneecap joint; (6) 20 cm proximal from the kneecap joint; and (7) the root of the leg, with volume calculated formula as previously described [11]. The ECW/TBW reflecting fluid imbalances due to lymphedema was calculated by bioelectrical impedance using a body composition analyzer, the InBody 720 or InBody 770. The reduction reflecting the effect of the treatment is defined as the value obtained by the following formula for each patient:

(1) $$\begin{array}{c} \text{reduction}=\left(\text{posttreatment value}\right)-\left(\text{pretreatment value}\right). \end{array}$$

### 2.5. Statistical Analysis

The distributions of patient characteristics were evaluated using the chi-squared test or Fisher's exact test for categorical variables. Statistical analysis was performed using Student's *t*-test, and the Wilcoxon signed-rank test was used for paired samples. Odds ratios with confidence intervals (CIs) were estimated by logistic regression. A probability (*P*) value <0.05 was considered to indicate statistical significance. All statistical analyses were conducted using JMP Pro 14.0.0 (SAS Institute Inc., Cary, NC).

## 3. Results

### 3.1. Patient Characteristics

Table 1 summarizes the patient characteristics of the 19 women who suffered from LLL after gynecologic cancer treatment, including pelvic lymphadenectomy. The mean age was 56.4 years (95% CI, 50.6–62.2). The median duration of the LLL diagnosis was 19.6 months after surgery (range 4.2–243,1 month). Ten patients (52.6%) were diagnosed with cervical cancer, seven patients (36.8%) with endometrial cancer, and one patient (10.5%) with ovarian cancer. Six patients (31.6%) received postoperative concurrent chemoradiotherapy with cisplatin plus 5-fluorouracil, nine patients (47.4%) received postoperative chemotherapy with paclitaxel plus carboplatin, and four patients (36.4%) received no adjuvant therapy. All patients received CDT, including SLD, compression therapy, and exercise. Eleven patients (63.6%) were treated with goreisan as a part of their treatment for LLL. The patients had a median age of 55.3 years in the CDT group and 57.3 years in the CDT-G group. There was no significant difference in patient characteristics between the two groups.

### 3.2. Pretreatment and Posttreatment Status of LLL

Table 2 shows the pretreatment status of the patients with LLL. The mean body mass index (BMI) and body weight (BW) in all patients were 22.59 kg/m^2^ (95% CI, 20.59–24.59) and 55.90 kg (95% CI, 50.76–61.04), respectively. The mean ECW/TBW values of the affected lower limb and unaffected lower limb were 0.404 and 0.394, respectively. No statistically significant difference was observed in the BMI, BW, ECW/TBW, and estimated volume of the patients in the CDT group and CDT-G group. Regarding the posttreatment status of LLL, there was no statistically significant difference in BMI, BW, ECW/TBW, and estimated volume between both groups (data are not shown).

### 3.3. Changes of Estimated Volume and ECW/TBW after Treatment

Table 3 summarizes the change in the severity of LLL after the intervention. For all patients, the mean reduction in their BMI and BW from the treatment was −0.24 kg/m^2^ and −0.62 kg, respectively. The mean reduction of the patients' ECW/TBW in the total body, affected lower limb, and unaffected lower limb was −0.0031, −0.0037, and −0.0025, respectively. The mean reduction in the estimated volume of the affected lower limb and unaffected lower limb was 15.73 and −63.63 mL, respectively. A comparison of the CDT group and the CDT-G group showed no statistical difference in the patients' BMI, BW, and estimated volume. However, there was a significant difference in the ECW/TBW of the total body and affected lower limbs between the two groups. No adverse events were observed during treatment among the patients in both groups.

## 4. Discussion

The purpose of this small-scale pilot study is to evaluate the effectiveness of goreisan combined with CDT on patients with LLL. Our study demonstrated that goreisan can alleviate lymphedema symptoms by improving systemic fluid retention. We also showed that the improvement in LLL could be detected more effectively by using ECW/TBW rather than the estimated lower-limb volume.

Our result suggests that combination therapy of CDT and goreisan is more effective than CDT alone. According to previous reports, polyporus and *Alismatis rhizoma*, among the ingredients contained in goreisan, are considered to be the main ingredients to promote diuresis and eliminate systemic water retention [12]. Ding et al. reported that goreisan significantly improves kidney function by reducing the creatinine levels in mice [8]. Another study demonstrated that the administration of goreisan could reduce BW in a nephrocalcinosis rat model [13]. The diuretic function of goreisan promotes the drainage of water retained in the interstitial space and may contribute to the improvement of LLL; however, the pharmacological activity of goreisan has not been fully elucidated at this point. Concerning the clinical use of goreisan, although several studies reported the clinical efficacy of goreisan on the prevention of the recurrence of chronic subdural hematoma and the improvement of postoperative nausea, there are only a few reports relating to goreisan's effect on lymphedema [14–16]. Komiyama et al. reported that goreisan might be effective on lower abdominal lymphedema following pelvic lymphadenectomy, though they did not provide a description of the combination therapy or have appropriate control cases [9]. To our best knowledge, a well-designed clinical trial has never confirmed the efficacy of goreisan in the treatment of LLL. This study is the first prospective study to verify the efficacy of goreisan on LLL. A randomized trial to evaluate the efficacy of goreisan for breast-cancer-related upper extremity lymphedema was launched in 2016; however, the results have not been published as yet [12]. Therefore, the position of goreisan in LLL treatment is currently controversial and inconsistent. Before launching a prospective randomized trial of goreisan treatment for LLL, we conducted this case series study to provide more reliable evidence of its efficacy. We are convinced that our results indicate the improved effect of goreisan on LLL in combination with CDT, which will lead to more verifiable studies. As the present study shows, the effect of goreisan is already evident in the first few weeks. Although the long-term efficacy is unclear, symptom improvement in the early stages of treatment can motivate patients to continue treatment. In Japan, it is approved to take 7.5 g of goreisan orally, divided into three times a day. Dose optimization based on body weight, renal function, or age is not necessary, and thus, we recommend the same dose of goreisan in the future study.

In this study, two different methods are used to quantify the severity of LLL. The edema-reducing effect of goreisan is clearer in ECW/TBW than in the estimated lower-limb volume based on the limb circumference measurements. Circumference measurement is the standard method for lymphedema evaluation because it is the most straightforward and economical method and has a strong correlation of actual volume measured by water displacement, [17, 18]. However, measurement errors based on limb circumference easily occur depending on the measurement time and the person who measures it, and therefore, treatment tends to be overestimated or underestimated. In our current study, we adopted bioelectrical impedance as another index reflecting the severity of LLL. Bioimpedance measurement is a noninvasive technology that can accurately measure a patient's TBW, ECW, and intracellular body water in clinical settings [19, 20]. Specifically, InBody is widely used for the diagnosis of lymphedema and monitoring limb edema [21, 22]. A recent study by Lim et al. demonstrated that bioelectrical impedance measured by InBody could be an alternative method to circumference measurement for monitoring breast-cancer-related lymphedema [23]. Our results are consistent with previous findings, supporting the evidence that bioelectrical impedance can be a standard method of measuring edema instead of circumference measurement.

In terms of drug therapy for lymphedema, diuretics have also been used for lymphedema in the hope of reducing edema. However, due to their adverse effects such as electrolyte imbalance, it is not recommended in most guidelines. In the present study, no apparent adverse effects were observed with the use of goreisan. Although adverse effects such as hepatic dysfunction and allergies have been reported in the past, goreisan is relatively safe to use in our experiences. A larger, prospective interventional trial will be needed to verify the safety of goreisan in patients with LLL.

The limitations of this study are the short period of evaluation and treatment. Moreover, the bias of clinicopathological features could not be eliminated because of the nonrandomized design. Also, the evaluators were not blinded to the treatment. The absence of a significant difference in the estimated lower-limb volume based on the limb circumference measurements may merely reflect the small number of patients, resulting in a type II error. Furthermore, there was a slight increase in estimated volume and ECW/TBW in patients in the CDT group. This result was unexpected and may be due to the fact that the difference between the pre and posttreatment interventions was too small to detect efficacy of CDT alone in patients with mild LLL. However, the strengths of our study include the prospective nature that enables us to equalize the quality and methodology of evaluation of LLL.

## 5. Conclusion

The present study is one of few demonstrating the treatment effectiveness of goreisan with CDT for LLL. We provide evidence that the administration of Japanese herbal medicine can improve the current standard of care for patients with LLL. In the future, it is necessary to conduct a randomized controlled trial to establish more reliable evidence.

## Figures and Tables

**Table 1:** Patient characteristics stratified by the CDT group and CDT-G group.

	Total (*n* = 19)	CDT group (*n* = 8)	CDT-G group (*n* = 11)	*P* value
Age (mean, 95% CI)	56.4 (50.6–62.2)	55.3 (46.1–64.5)	57.3 (49.4–65.1)	0.737
Duration to diagnosis (median, range)	19.5 (4.2–243.1)	40.1 (4.4–243.1)	10.7 (4.2–162.1)	0.386
Body mass index (mean, 95% CI)	22.6 (20.6–24.6)	23.3 (20.2–26.5)	22.1 (19.4–24.8)	0.535
Type of cancer (*n*, (%))				0.965
Cervical cancer	10 (52.6)	4 (50.0)	6 (54.5)	
Endometrial cancer	7 (36.8)	3 (37.5)	4 (36.4)	
Ovarian cancer	2 (10.5)	1 (12.5)	1 (9.1)	
Type of lymphadenectomy (*n*, (%))				0.830
PLN	16 (84.2)	7 (87.5)	9 (81.8)	
PLN + PAN	3 (15.8)	1 (12.5)	2 (18.2)	
Chemotherapy (*n*, (%))				0.425
Yes	15 (78.9)	7 (87.5)	8 (72.7)	
No	4 (21.1)	1 (12.5)	3 (27.3)	
Radiotherapy (*n*, %)				0.637
Yes	6 (31.6)	3 (37.5)	3 (27.3)	
No	13 (68.4)	5 (62.5)	8 (72.7)	
Affected side (*n*, (%))				0.255
Left	9 (47.4)	5 (62.5)	4 (36.4)	
Right	10 (52.6)	3 (37.5)	7 (63.6)	

CDT, complex decongestive therapy; CDT-G, combination of complex decongestive therapy and goreisan; CI, confidence interval; PLN, pelvic lymphadenectomy; PAN, paraaortic lymphadenectomy.

**Table 2:** Pre and posttreatment status of LLL stratified by the CDT group and CDT-G group.

	Total population (*n* = 19)	CDT group (*n* = 8)	CDT-G group (*n* = 11)	*P* value
Pretreatment status				
BMI (kg/m^2^) (mean, 95% CI)	22.59 (20.59–24.59)	23.31 (20.17–26.47)	22.08 (19.39–24.76)	0.5346
Body mass (kg) (mean, 95% CI)	55.90 (50.76–61.04)	57.55 (49.44–65.67)	54.71 (47.79–61.63)	0.5814
ECW/TBW (mean, 95% CI)				
Total body	0.396 (0.390–0.401)	0.392 (0.384–0.400)	0.398 (0.392–0.405)	0.2292
Affected lower limb	0.404 (0.394–0.413)	0.399 (0.384–0.413)	0.407 (0.395–0.419)	0.34
Unaffected lower limb	0.394 (0.390–0.399)	0.391 (0.384–0.398)	0.397 (0.391–0.403)	0.2072
Estimated volume (mL) (mean, 95% CI)				
Affected lower limb	7134.54 (6235.6–8033.5)	7529.88 (6123.2–8936.5)	6847.02 (5647.4–8046.6)	0.4465
Unaffected lower limb	6454.25 (5760.5–7148.0)	6818.64 (5741.3–7896.0)	6189.24 (5270.5–7108.0)	0.3614
Posttreatment status				
BMI (kg/m^2^) (mean, 95% CI)	22.36 (20.33–24.39)	23.51 (20.37–26.65)	21.53 (18.85–24.21)	0.3253
Body mass (kg) (mean, 95% CI)	55.27 (50.26–60.29)	58.01 (50.28–65.78)	53.29 (46.67–59.91)	0.3427
ECW/TBW (mean, 95% CI)				
Total body	0.393 (0.388–0.397)	0.392 (0.385–0.399)	0.393 (0.387–0.399)	0.8366
Affected lower limb	0.400 (0.392–0.408)	0.399 (0.387–0.412)	0.400 (0.390–0.411)	0.8976
Unaffected lower limb	0.392 (0.387–0.396)	0.391 (0.384–0.398)	0.392 (0.386–0.399)	0.733
Estimated volume (mL) (mean, 95% CI)				
Affected lower limb	7150.27 (6210.2–8090.3)	7671.53 (6216.1–9127.0)	6771.18 (5530.0–8012.4)	0.3346
Unaffected lower limb	6390.62 (5746.5–7034.8)	6688.24 (5682.1–7694.3)	6174.17 (5316.2–7032.2)	0.4234

LLL, lower-limb lymphedema; CDT, complex decongestive therapy; CDT-G, combination of complex decongestive therapy and goreisan; BMI, body mass index; CI, confidence interval; ECW/TBW, ratio of extracellular water to total body water.

**Table 3:** Improvement in status of LLL from pretreatment to posttreatment.

Reduction	Total population (*n* = 19)	CDT group (*n* = 8)	CDT-G group (*n* = 11)	*P* value
BMI (kg/m^2^) (mean, 95% CI)	−0.24 (−0.85 to 0.38)	0.19 (−0.74 to 1.12)	−0.55 (−1.34 to 0.24)	0.2183
Body mass (kg) (mean, 95% CI)	−0.62 (−2.22 to 0.97)	0.46 (−1.98 to 2.90)	−1.42 (−3.50 to 0.66)	0.2331
ECW/TBW (mean, 95% CI)				
Total body	−0.0031 (−0.0059 to −0.0004)	−0.0001 (−0.0039 to 0.0037)	−0.0053 (−0.0086 to −0.0021)	0.0417
Affected lower limb	−0.0037 (−0.0077 to 0.0003)	0.0007 (−0.0041 to 0.0056)	−0.0069 (−0.0126 to −0.0012)	0.0423
Unaffected lower limb	−0.0025 (−0.0061 to 0.0010)	−0.0001 (−0.0055 to 0.0052)	−0.0042 (−0.0089 to 0.0003)	0.2324
Estimated volume (mL) (mean, 95% CI)				
Affected lower limb	15.73 (−245.27 to 276.73)	141.65 (−265.3 to 548.57)	−75.84 (−422.9 to 271.18)	0.4028
Unaffected lower limb	−63.63 (−313.92 to 186.66)	−130.40 (−526.5 to 265.65)	−15.07 (−352.8 to 322.69)	0.6461

LLL, lower-limb lymphedema; CDT, complex decongestive therapy; CDT-G, combination of complex decongestive therapy and goreisan; BMI, body mass index; CI, confidence interval; ECW/TBW, ratio of extracellular water to total body water.

## Data Availability

The datasets used and/or analysed during the current study are available from the corresponding author upon reasonable request.
